# Postoperative Thromboembolism According to the Type of Surgery: A Nationwide Study in the Republic of Korea

**DOI:** 10.3390/jcm11061477

**Published:** 2022-03-08

**Authors:** Ka-Won Kang, Ji Yoon Lee, Byung-Hyun Lee, Min Ji Jeon, Eun Sang Yu, Dae Sik Kim, Se Ryeon Lee, Chul Won Choi, Yong Park, Hwa Jung Sung, Byung Soo Kim

**Affiliations:** 1 Division of Hematology-Oncology, Department of Internal Medicine, Korea University College of Medicine, Seoul 02841, Korea; ggm1018@gmail.com (K.-W.K.); potato0430@hanmail.net (B.-H.L.); tinymj@naver.com (M.J.J.); imdryu@gmail.com (E.S.Y.); kay9801@naver.com (D.S.K.); logost@hanmail.net (S.R.L.); bonnie@korea.ac.kr (C.W.C.); paark76@hanmail.net (Y.P.); 2 Department of Biostatistics, Korea University College of Medicine, Seoul 02841, Korea; jinny1502@korea.ac.kr; 3 Transdisciplinary Major in Learning Health Systems, Department of Healthcare Sciences, Graduate School, Korea University, Seoul 02841, Korea

**Keywords:** thromboembolism, incidence, surgical procedures

## Abstract

Postoperative thromboembolism (TE) is a serious, but preventable, complication in surgical patients. Orthopedic surgery, neurosurgery, and vascular surgery are considered high risk for TE, and current guidelines recommend TE prophylaxis. However, insufficient data exist regarding TE risk in other general surgeries. This study identified the actual incidence and relative risk of postoperative TE in the real world, according to surgery type. Twenty-six surgeries between 1 December 2017 and 31 August 2019 were selected from the Health Insurance Review and Assessment Service database and analyzed for postoperative TE events. Among all patients, 2.17% had a TE event within 6 months of surgery and 0.75% had a TE event owing to anticoagulant treatment. The incidence of total TE events was the highest in total knee replacement (12.77%), hip replacement (11.46%), and spine surgery (5.98%). The incidence of TE with anticoagulant treatment was the highest in total knee replacement (7.40%), hip replacement (7.20%), and coronary artery bypass graft (CABG) surgery (3.81%). Hip replacement, total knee replacement, CABG surgery, spine surgery, and cardiac surgery except CABG surgery, showed relatively higher risks for total claimed venous TE. The relative risk of venous TE with anticoagulant treatment was the highest for hysterectomy, partial hepatectomy, hip replacement, cardiac surgery except CABG surgery, and total knee replacement. The relative risk of arterial TE was the highest for cardiac surgery, total knee replacement, and hip replacement. In the real world, the incidence of postoperative TE events from total knee replacement and those from hip replacement remain high, and some surgeries could have a relatively higher risk of TE than other surgeries. For patients undergoing these surgeries, studies to reduce the incidence of postoperative TE in clinical practice should be conducted.

## 1. Introduction

Postoperative thromboembolism (TE) is a serious, but preventable, complication in patients undergoing surgery. Three factors affect the development of TE: vascular injury, impairment of blood flow, and hypercoagulability [1]. To reduce the incidence of postoperative TE, surgical patients may be advised early walking, use of sequential compression devices, and antiplatelet or anticoagulant treatment. This is more apparent in patients who are particularly at risk for TE, namely those with cancer, atrial fibrillation, atrial flutter, or history of TE, as well as those scheduled to undergo surgeries that are known to be high risk for TE [2,3,4,5].

Surgery, in itself, increases the risk of TE, but some surgeries are categorized as having a high risk of postoperative TE [6,7]. Historically, these surgeries include orthopedic surgery, neurosurgery, and vascular surgery, and current guidelines recommend mechanical and pharmacological prophylaxis for TE in patients undergoing such procedures [5,8,9]. In the case of other general surgeries, patients are assessed for postoperative TE risk, either clinically or with the help of more objective measures such as the Caprini risk assessment model or the Rogers score [10,11]. The benefit of adding pharmacological prophylaxis to counteract the risk of bleeding is then left to the discretion of the physician [9,11,12,13,14,15]. Although objective measures exist, the risk for TE is mainly judged based on patient characteristics, and there are limited data on the extent of postoperative TE risk according to the type of surgery. In particular, there are limited data on TE risk in the Asian population, wherein TE risk is generally thought to be lower than that in Western countries.

Using a nationally representative database, this study aimed to identify the real-world incidence and pattern of postoperative TE in patients who had undergone commonly practiced surgeries and assess the relative risk for TE according to the type of surgery.

## 2. Materials and Methods

### 2.1. Data Source

In the Republic of Korea, the National Health Insurance (NHI), a universal health coverage system, covers approximately 98% of the population. NHI pays medical costs based on claims data, which consists of diagnosis, treatment, procedures, and drug prescriptions [16,17]. The claims data are anonymized and made available for healthcare service research in the Health Insurance Review and Assessment Service (HIRA) database, which was used for this study. All procedures involving human participants in this study were performed in accordance with the ethical standards of the institutional and national research committees and in accordance with the 1964 Helsinki Declaration and its later amendments or comparable ethical standards. The study was approved by the institutional review board of the Korea University Anam Hospital (No. 2020AN0136). The requirement for informed consent was waived by the institutional review board of the Korea University Anam Hospital, owing to the use of anonymized patient data.

### 2.2. Patients

The 2019 Statistical Information Report for Major Surgical Statistics provided by the National Statistical Office was used to select surgeries commonly performed in the Republic of Korea (http://kostat.go.kr/, accessed on 1 April 2020). The report consisted of data on 33 surgeries, including 16 surgeries of international interest and 17 surgeries reflecting domestic conditions. Among them, 26 surgeries that either required general anesthesia or were considered commonly performed procedures were selected for the present study. 

We extracted all data from the HIRA database of each patient who had undergone the selected surgeries between 1 December 2017 and 31 August 2019 and analyzed TE events of these patients from the date of surgery up to 6 months (Table S1). 

### 2.3. Clinical Endpoints

The primary endpoints were the incidence of postoperative TE and its relative risk according to the type of surgery. ‘Postoperative TE events’ was defined as (1) patients with any venous and arterial TE events that were claimed within 6 months from the date of the surgery (total claimed TE); and (2) patients with venous and arterial TE events that were claimed within 6 months from the date of the surgery and treated with anticoagulant medications within 3 days of the date of TE diagnosis (TE with anticoagulant treatment).

‘The patients who underwent prophylactic anticoagulant treatment’ were defined as patients satisfying the following conditions, to distinguish them from those using the anticoagulant treatment for therapeutic purposes: (1) patients who were administered anticoagulant medications within 3 days of the date of the surgery, among those who had no TE events; or (2) patients who were administered anticoagulant medications from the day of surgery to 1 day before the diagnosis of TE events among those who had TE events.

The risk factors for TE included age, sex, history of cancer, atrial fibrillation or atrial flutter, history of TE, history of taking antiplatelet or anticoagulant drugs between 2 weeks and 3 months before surgery, antiplatelet or anticoagulant use on the day of surgery, and use of prophylactic anticoagulant treatment. The claims data for cancer, atrial fibrillation or atrial flutter, and TE diagnosis were limited to within 3 months from the date of surgery. Antiplatelet drugs included aspirin, clopidogrel, cilostazol, and ticagrelor, and anticoagulant drugs included apixaban, edoxaban, dabigatran, argatroban, enoxaparin, dalteparin, heparin, and warfarin.

### 2.4. Statistical Analysis

Continuous data are presented as means and standard deviations, and categorical data are presented as frequencies and percentages. The incidence of TE according to each surgery was calculated as the number of patients who met the definition for TE mentioned above. The adjusted odds ratios (ORs) for TE events were obtained using a multiple logistic regression model, with the following independent variables: age, sex, history of cancer, atrial fibrillation or atrial flutter, TE, antiplatelet or anticoagulant drug use between 2 weeks and 3 months before surgery and on the day of surgery, and use of prophylactic anticoagulant treatment. The multivariable analysis results are presented as adjusted ORs and their 95% confidence intervals (CIs). Transurethral prostatectomy was used as a reference category for the types of surgery. All statistical analyses were performed using SAS version 9.4 software (SAS Institute Inc., Cary, NC, USA).

## 3. Results

### 3.1. Incidence of TE within 6 Months from the Date of Surgery

Data of a total of 2,799,293 patients who underwent the selected surgeries between 1 December 2017 and 31 August 2019 were analyzed. Among those patients, 60,683 (2.17%) had a TE event within 6 months from the date of surgery and 21,063 (0.75%) had a TE event following anticoagulant treatment (Table 1). With respect to TE in the anticoagulant treatment group, the median duration of anticoagulant therapy was 13 days (range: 1–442 days).

**Figure 1:**
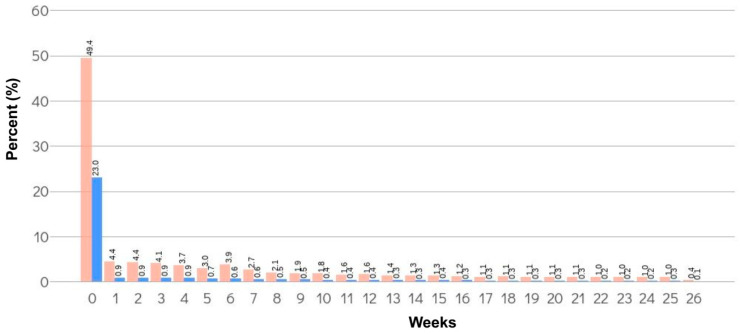
Distribution of TE events within 6 months from the date of surgery. The distribution of TE events within 6 months from the date of surgery is shown as the percentage from a total of 60,683 patients with TE (pink bar: total claimed TE, blue bar: TE with anticoagulant treatment).

The incidence of total claimed TE events was the highest for total knee replacement (13,811/108,111 patients, 12.77%), hip replacement (4975/43,415 patients, 11.46%), and spine surgery (15,857/265,317 patients, 5.98%). In the case of TE in the anticoagulant treatment, the incidence was the highest in total knee replacement (7995/108,111 patients, 7.40%), hip replacement (3124/43,415 patients, 7.20%), and coronary artery bypass graft surgery (251/6591 patients, 3.81%). Approximately 65% of TE events occurred within 1 month of surgery (Figure 1).

### 3.2. Proportion of Patients Who Underwent Prophylactic Anticoagulant Treatment and Its Effect on the Incidence of TE Events

Among 2,799,293 patients enrolled in this study, 158,987 patients underwent prophylactic anticoagulant treatment (mean: 5.68%, range: 0.06–96.77% according to the type of surgery). The proportions of patients who underwent prophylactic anticoagulant treatment for each type of surgery were 52.62% in total knee replacement, 41.17% in hip replacement, 9.06% in spine surgery, 40.77% in partial hepatectomy, 26.18% in gastrectomy, and 18.96% in hysterectomy. The incidence of postoperative TE events from prophylactic anticoagulant treatment was approximately 90% lower than that with no treatment (Table 2). 

### 3.3. Type of TE within 6 Months from the Date of Surgery

The types of total claimed TE and TE with anticoagulant treatment are summarized in Table 3. In the total claimed TE, 79.78% (48,412/60,683 patients) of patients were diagnosed with venous TE and 20.22% (12,271/60,683 patients) with arterial TE. In the cases where the location was identified, the lower extremities were the most common location for venous TE (18,690/48,410 patients, 38.61%). For arterial TE, the extremities in general were the most common location (1780/12,273 patients, 14.50%). In TE with anticoagulant treatment, 91.27% (19,224/60,683 patients) of patients were diagnosed with venous TE and 8.73% (1839/60,683 patients) with arterial TE. Myocardial infarction occurred in 0.023% of patients (14/60,683 patients), stroke in 0.089% (54/60,683 patients), and pulmonary embolism in 0.003% (2/60,683 patients). The types of total claimed TE and TE with anticoagulant treatment according to each surgery type are presented in Tables S2 and S3.

### 3.4. Relative risk of TE According to Surgery Type

To analyze the relative risk of TE for each surgery, the adjusted ORs for TE events were calculated, after correcting for TE risk factors. In this study, 2.84% of patients (79,498/2,799,293 patients) had a history of cancer, 0.002% (48/2,799,293 patients) had atrial fibrillation or atrial flutter, 0.44% (12,420/2,799,293 patients) had a history of TE, 11.11% (310,994/2,799,293 patients) were taking antiplatelet or anticoagulant drugs between 2 weeks and 3 months prior to surgery, 7.03% (196,745/2,799,293 patients) were taking antiplatelet or anticoagulant drugs on the day of surgery, and 5.68% (158,987/2,799,293 patients) underwent prophylactic anticoagulant treatment. The baseline characteristics (whole cohort and each surgery group) and the ORs and 95% CI of each risk factor are presented in Tables S4 and S5. Transurethral prostatectomy was used as a reference surgery.

**Figure 2:**
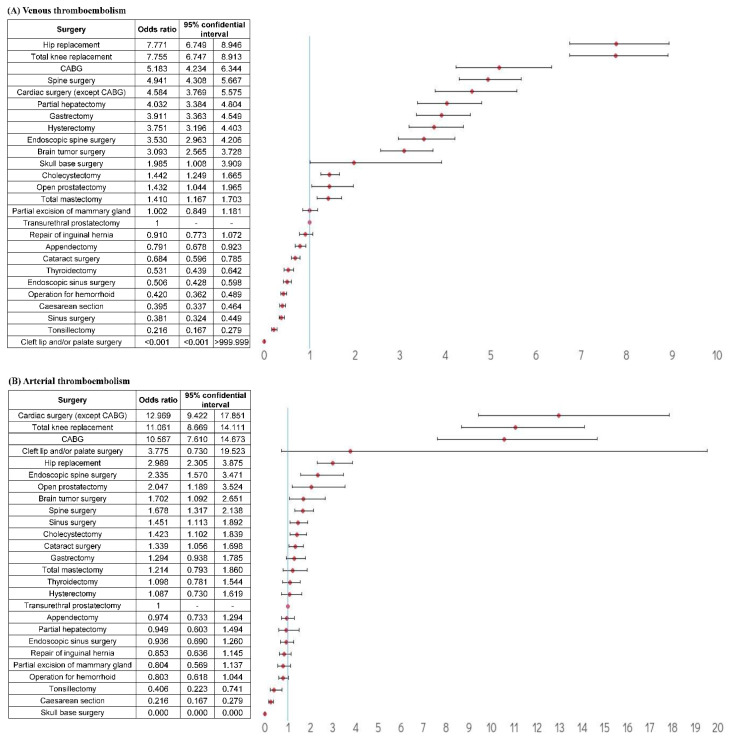
Odds ratios of total claimed thromboembolism events for each type of surgery after correcting for thromboembolism risk factors. Abbreviation: CABG, coronary artery bypass graft. (**A**) The total claimed venous thromboembolism events, (**B**) the total claimed arterial thromboembolism events.

The relative risk of the total claimed venous TE for each surgery, after correcting for TE risk factors, revealed that the highest risk was associated with hip replacement (OR = 7.771, 95% CI: 6.749–8.946), total knee replacement (OR = 7.755, 95% CI: 6.747–8.913), and coronary artery bypass graft surgery (OR = 5.183, 95% CI: 4.234–6.344), followed by spine surgery (OR = 4.941, 95% CI: 4.308–5.667), cardiac surgery (except for coronary artery bypass graft surgery) (OR = 4.584, 95% CI: 3.769–5.575), partial hepatectomy (OR = 4.032, 95% CI: 3.384–4.804), gastrectomy (OR = 3.911, 95% CI: 3.363–4.549), and hysterectomy (OR = 3.751, 95% CI: 3.196–4.403) (Figure 2A). The relative risk of the total claimed arterial TE for each surgery after correcting for TE risk factors revealed that the highest risk was associated with cardiac surgery (except for coronary artery bypass graft surgery) (OR = 12.969, 95% CI: 9.422–17.851), total knee replacement (OR = 11.061, 95% CI: 8.669–14.111), and coronary artery bypass graft surgery (OR = 10.567, 95% CI: 7.610–14.673) (Figure 2B).

**Figure 3:**
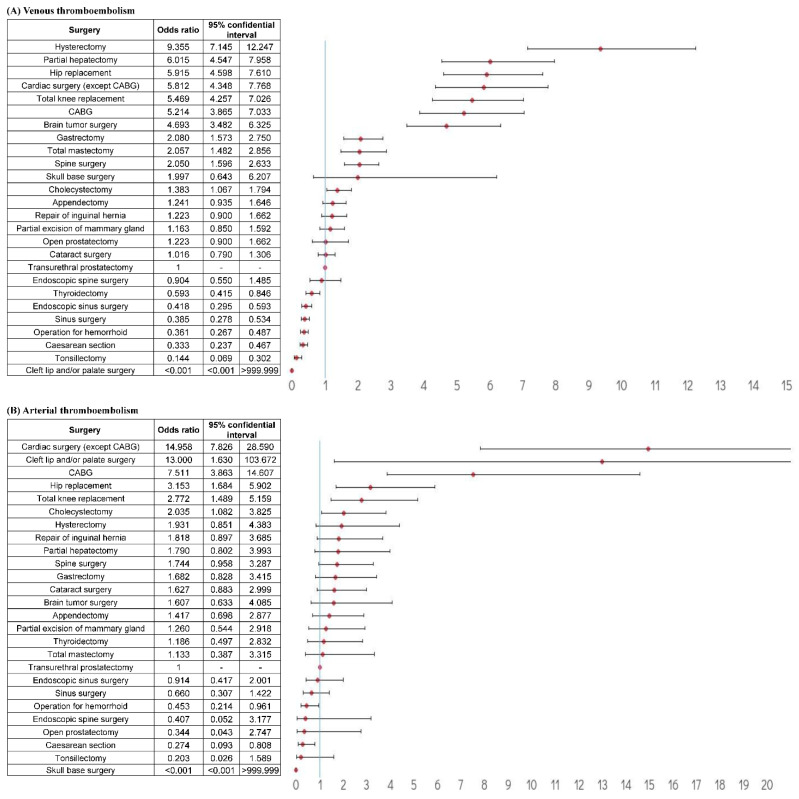
Odds ratios of thromboembolism events with anticoagulant treatment for each type of surgery after correcting for thromboembolism risk factors. Abbreviation: CABG, coronary artery bypass graft. (**A**) venous thromboembolism events with anticoagulant treatment (**B**) arterial thromboembolism events with anticoagulant treatment.

The relative risk of venous TE with anticoagulant treatment for each surgery after correcting for TE risk factors revealed that the highest risk was associated with hysterectomy (OR = 9.355; 95% CI: 7.145–12.247), partial hepatectomy (OR = 6.015; 95% CI: 4.547–7.958), and hip replacement (OR = 5.915, 95% CI: 4.598–7.610), followed by cardiac surgery (except for coronary artery bypass graft surgery) (OR = 5.812, 95% CI: 4.348–7.768), total knee replacement (OR = 5.469, 95% CI: 4.257–7.026), coronary artery bypass graft surgery (OR = 5.214, 95% CI: 3.865–7.033), and brain tumor surgery (OR = 4.693, 95% CI: 3.482–6.325) (Figure 3A). The relative risk of arterial TE with anticoagulant treatment for each surgery after correcting for the TE risk factors revealed that the highest risk was associated with cardiac surgery (except for coronary artery bypass graft surgery) (OR = 14.958, 95% CI: 7.826–28.590), cleft lip and/or palate surgery (OR = 13.000, 95% CI: 1.630–103.672), and coronary artery bypass graft surgery (OR = 7.511, 95% CI: 3.863–14.607) (Figure 3B).

## 4. Discussion

In this study of 2,799,293 patients who underwent selected surgeries from 1 December 2017 to 31 August 2019 in the Republic of Korea, 60,683 patients had claimed TE events (average: 2.17%, and range according to surgery type: 0.14–12.77%) and 21,063 had a TE event with anticoagulant treatment (average: 0.75%, and range according to surgery type: 0.01–7.40%). Approximately 65% of TE events occurred within 1 month of surgery, and the incidence of TE events differed according to the surgery type. Patients undergoing total knee replacement and hip replacement, i.e., 12.77% (13,811 of 108,111 patients) and 11.46% (4975 of 43,415 patients), respectively, had a higher incidence of total claimed TE. In addition, the relative risk of venous TE in these surgeries was also higher than that in other procedures. Several surgeries, including hysterectomy, partial hepatectomy, and gastrectomy, also showed a relatively higher risk of venous TE. The relative risk of arterial TE was the highest in cardiac surgery, total knee replacement, and hip replacement.

In Western countries, the incidence of postoperative TE events in patients undergoing total knee or hip replacement and spine surgery without TE prophylaxis ranges from 40% to 60% and 15% to 40%, respectively [18,19,20]. In Asian populations, the incidence of TE events reportedly ranges from 10% to 60% in total knee or hip replacement surgeries and approximately 30% in spine surgery, which is relatively lower than the rates in the Western population [21,22,23]. When pharmacological prophylaxis is added, postoperative TE events can be reduced by approximately 70–90% or more [24,25,26]. In this study, the incidence of total claimed TE in total knee replacement, hip replacement, and spine surgery was 12.77%, 11.46%, and 5.98%, respectively, which are higher incidence rates than those reported in previous studies [18,19,20,21,22,23,24,25,26]. Even considering the incidence of TE with anticoagulant treatment, total knee replacement and hip replacement were associated with a higher incidence of 7.4% and 7.2%, respectively. There are several possible explanations for these differences. First, previous studies focused on reporting the incidence of deep vein thrombosis of the lower extremities or pulmonary embolism. However, this study reported all TE events in the real world, including venous TE and arterial TE, using claims data. Second, adherence to postoperative TE prophylaxis, especially pharmacological prophylaxis, is usually lower in clinical practice than the guideline recommendations according to patients’ condition or the guidelines of each institution [27,28,29]. In fact, in this study, the rates of pharmacological prophylaxis were 52.62% and 41.17% in total knee replacement and hip replacement surgery, respectively, which are generally recommended for pharmacological prophylaxis. In addition, in the patient group that underwent pharmacological prophylaxis, it was confirmed that the incidence of postoperative TE events was reduced by approximately 90% compared with that in the no treatment group, as demonstrated in previous studies [24,25,26]. Taken together, in the real world, the incidence of postoperative TE may appear higher than that expected in previous studies because of low compliance with pharmacological prophylaxis.

Among all claimed TE events, TE events with anticoagulant treatment warranty the attention of physicians, excluding cases of superficial TE or subclinical TE events (Table 1). However, in the case of patients undergoing cardiac surgery, in which postoperative anticoagulant treatment is used in most patients, TE events with anticoagulant treatment may be overestimated. Conversely, in spine surgery, where the use of anticoagulant treatment is often difficult, because of the risk of bleeding, TE events with anticoagulant treatment in these patients may be underestimated. Therefore, this study presented both the total claimed TE, and TE with anticoagulant treatment, as the primary endpoints. However, the duration of anticoagulant treatment was shorter than that in the recommended guidelines for patients with postoperative TE events (median, 13 days; range, 1–442 days) [30,31]. This may be because of poor compliance with postoperative TE treatment guidelines, or patients using anticoagulant drugs for reasons other than postoperative TE treatment may have been included in the analysis. Thus, proper interpretation is necessary, to consider this aspect when applying the results of this study to clinical practice.

This study had the following differences compared to previous studies. First, as mentioned above, in most studies, TE events were counted by focusing on pulmonary embolism and deep vein thrombosis of the lower extremities [32,33]. Although pulmonary embolism and deep vein thrombosis of the extremities are clinically meaningful, in the real world, TE may occur in rare locations, such as intra-abdominally; and arterial TE, such as myocardial infarction and stroke, may also occur. In this study, we presented the situation of all TE events that can occur in the real world. Second, existing studies reported the risk of TE limited to one-area surgery or cancer-related surgeries [32,33,34,35]. Based on this information, it is impossible to sufficiently explain the relative risk for TE in each type of surgery in patients undergoing that surgery. Unlike other studies, this study aimed to present the relative risk for TE according to the type of surgery in patients who underwent commonly performed surgeries in clinical practice. Third, this study also presented the incidence of arterial TE in the real world. Although the rate was lower than that of venous TE, fatal arterial TE, such as that resulting in postoperative myocardial infarction or stroke, can occur [36,37,38,39]. However, there are insufficient data on the incidence of arterial TE in surgical patients in the real world, and this study manifested this fact.

This study had several limitations. First, since the analysis used claims data, there were limitations in identifying all TE risk factors, such as information on individual and family medical history related to thrombophilia and detailed laboratory tests. Furthermore, it was not possible to correct for cases of mechanical heart valve surgery as a major TE risk factor, because we analyzed patients who underwent each selected surgery for the first time during the study period. However, the proportion of patients who had undergone mechanical heart valve surgery among all the patients was 0.002% (5921/2,799,293 patients); therefore, the effect of this was small. Second, because details such as the patient’s condition at the time of each surgery, purpose of the surgery, and situation at the time of the surgery were unknown, other factors in each surgery that were associated with an increased relative risk of TE could not be analyzed. Nevertheless, this study is considered to be meaningful because it presents the incidence and pattern of all postoperative venous or arterial TE of the commonly practiced surgeries in the real world and relative risk according to the type of surgery, using a nationally representative database.

In conclusion, in the real world, the incidence of postoperative venous TE in total knee replacement and hip replacement is still high, and some surgeries could have a relatively higher risk of venous TE than other surgeries. For patients undergoing these surgeries, studies to reduce the incidence of postoperative TE in clinical practice should be conducted.

## Figures and Tables

**Table 1:** Incidence of thromboembolism within 6 months from the date of each surgery.

Surgery	Total Patients, *n*	Thromboembolism within 6 Months from the Date of Surgery		
		A: Total Claimed Thromboembolism, *n* (%)	B: Thromboembolism with Anticoagulant Treatment, *n* (%)	B/A (%) *
Total knee replacement	108,111	13,811 (12.77)	7995 (7.40)	57.89
Hip replacement	43,415	4975 (11.46)	3124 (7.20)	62.79
Spine surgery	265,317	15,857 (5.98)	3336 (1.26)	21.04
Coronary artery bypass graft	6591	343 (5.20)	251 (3.81)	73.18
Partial hepatectomy	9322	429 (4.60)	331 (3.55)	77.16
Cardiac surgery (except coronary artery bypass graft)	10,296	443 (4.30)	386 (3.75)	87.13
Gastrectomy	27,203	1114 (4.10)	302 (1.11)	27.11
Hysterectomy	20,831	714 (3.43)	540 (2.59)	75.63
Endoscopic spine surgery	12,157	392 (3.22)	23 (0.19)	5.87
Brain tumor surgery	11,516	302 (2.62)	173 (1.50)	57.28
Open prostatectomy	3387	81 (2.39)	23 (0.68)	28.40
Cholecystectomy	129,081	2511 (1.95)	828 (0.64)	32.97
Transurethral prostatectomy	18,547	342 (1.84)	85 (0.46)	24.85
Skull base surgery	674	10 (1.48)	4 (0.59)	40.00
Cataract surgery	907,397	12,819 (1.41)	2489 (0.27)	19.42
Total mastectomy	22,091	280 (1.27)	98 (0.44)	35.00
Repair of inguinal hernia	56,698	671 (1.18)	153 (0.27)	22.80
Partial excision of mammary gland	63,033	586 (0.93)	130 (0.21)	22.18
Appendectomy	137,344	1024 (0.75)	263 (0.19)	25.68
Thyroidectomy	51,506	351 (0.68)	80 (0.16)	22.79
Sinus surgery	158,677	867 (0.55)	82 (0.05)	9.46
Endoscopic sinus surgery	109,454	578 (0.53)	115 (0.11)	19.90
Operation for hemorrhoid	303,456	1401 (0.46)	158 (0.05)	11.28
Cesarean section	255,600	684 (0.27)	84 (0.03)	12.28
Cleft lip and/or palate surgery	1282	2 (0.16)	1 (0.08)	50.00
Tonsillectomy	66,307	96 (0.14)	9 (0.01)	9.38
Total	2,799,293	60,683 (2.17)	21,063 (0.75)	34.71

Note: * B/A represents the ratio of thromboembolism events with anticoagulant treatment from the total claimed thromboembolism events.

**Table 2:** Proportion of patients who underwent prophylactic anticoagulant treatment and its effect on the incidence of TE events.

Surgery	Total Patients, *n*	Patients Who Underwent Prophylactic Anticoagulant Treatment, *n* (%)	Total Claimed Thromboembolism, *n* (%)			Thromboembolism with Anticoagulant Treatment, *n* (%)		
			With Prophylactic Anticoagulant Treatment	Without Prophylactic Anticoagulant Treatment	*p* Value	With Prophylactic Anticoagulant Treatment	Without Prophylactic Anticoagulant Treatment	*p* Value
Total knee replacement	108,111	56,887 (52.62)	1717 (1.59)	12,094 (11.19)	<0.001	551 (0.51)	7444 (6.89)	<0.001
Hip replacement	43,415	17,876 (41.17)	684 (1.58)	4291 (9.88)	<0.001	350 (0.81)	2774 (6.39)	<0.001
Spine surgery	265,317	24,029 (9.06)	1131 (0.43)	14,726 (5.55)	<0.001	551 (0.21)	2785 (1.05)	<0.001
Coronary artery bypass graft	6591	6378 (96.77)	162 (2.46)	181 (2.75)	0.305	70 (1.06)	181 (2.75)	<0.001
Partial hepatectomy	9322	3801 (40.77)	134 (1.44)	295 (3.16)	<0.001	94 (1.01)	237 (2.54)	<0.001
Cardiac surgery (except coronary artery bypass graft)	10,296	9799 (95.17)	216 (2.10)	227 (2.20)	0.601	161 (1.56)	225 (2.19)	0.001
Gastrectomy	27,203	7123 (26.18)	164 (0.60)	950 (3.49)	<0.001	79 (0.29)	223 (0.82)	<0.001
Hysterectomy	20,831	3949 (18.96)	178 (0.85)	536 (2.57)	<0.001	126 (0.60)	414 (1.99)	<0.001
Endoscopic spine surgery	12,157	86 (0.71)	3 (0.02)	389 (3.20)	<0.001	1 (0.01)	22 (0.18)	<0.001
Brain tumor surgery	11,516	4888 (42.45)	126 (1.09)	176 (1.53)	0.004	75 (0.65)	98 (0.85)	0.0804
Open prostatectomy	3387	1708 (50.43)	44 (1.30)	37 (1.09)	0.437	9 (0.27)	14 (0.41)	0.2971
Cholecystectomy	129,081	10,164 (7.87)	356 (0.28)	2155 (1.67)	<0.001	202 (0.16)	626 (0.48)	<0.001
Transurethral prostatectomy	18,547	678 (3.66)	41 (0.22)	301 (1.62)	<0.001	24 (0.13)	61 (0.33)	<0.001
Skull base surgery	674	258 (38.28)	6 (0.89)	4 (0.59)	0.527	2 (0.30)	2 (0.30)	1.000
Cataract surgery	907,397	2432 (0.27)	760 (0.08)	12,059 (1.33)	<0.001	460 (0.05)	2029 (0.22)	<0.001
Total mastectomy	22,091	2278 (10.31)	66 (0.30)	214 (0.97)	<0.001	33 (0.15)	65 (0.29)	0.001
Repair of inguinal hernia	56,698	559 (0.99)	54 (0.10)	617 (1.09)	<0.001	25 (0.04)	128 (0.23)	<0.001
Partial excision of mammary gland	63,033	1341 (2.13)	68 (0.11)	518 (0.82)	<0.001	37 (0.06)	93 (0.15)	<0.001
Appendectomy	137,344	1291 (0.94)	88 (0.06)	936 (0.68)	<0.001	58 (0.04)	205 (0.15)	<0.001
Thyroidectomy	51,506	1372 (2.66)	40 (0.08)	311 (0.60)	<0.001	22 (0.04)	58 (0.11)	<0.001
Sinus surgery	158,677	275 (0.17)	39 (0.02)	828 (0.52)	<0.001	17 (0.01)	98 (0.06)	<0.001
Endoscopic sinus surgery	109,454	801 (0.73)	32 (0.03)	546 (0.50)	<0.001	14 (0.01)	68 (0.06)	<0.001
Operation for hemorrhoid	303,456	179 (0.06)	34 (0.01)	1367 (0.45)	<0.001	18 (0.01)	140 (0.05)	<0.001
Cesarean section	255,600	690 (0.27)	16 (0.01)	668 (0.26)	<0.001	10 (0.003)	74 (0.03)	<0.001
Cleft lip and/or palate surgery	1282	5 (0.39)	-	2 (0.16)	-	0 (0.00)	1 (0.08)	-
Tonsillectomy	66,307	140 (0.21)	7 (0.01)	89 (0.13)	<0.001	4 (0.01)	5 (0.01)	0.739
Total	2,799,293	158,987 (5.68)	6166 (0.22)	4517 (1.95)	<0.001	2993 (0.11)	18,070 (0.65)	<0.001

**Table 3:** Type of TE within 6 months from the date of each surgery.

Type of TE	No.	Details	Total Claimed TE (*n* = 60,683)		TE with Anticoagulant Treatment (*n* = 21,603)	
			Number of Patients, *n*	Events (%)	Number of Patients, *n*	Events (%)
Venous TE	1	I26 Pulmonary embolism	2	0.003%	2	0.009%
	2	I80 Phlebitis and thrombophlebitis	5	0.008%	-	0.000%
	3	I80.0 Phlebitis and thrombophlebitis of superficial vessels of lower extremities	884	1.457%	147	0.698%
	4	I80.1 Phlebitis and thrombophlebitis of femoral vein	87	0.143%	31	0.147%
	5	I80.2 Phlebitis and thrombophlebitis of other deep vessels of lower extremities	13,931	22.957%	7717	36.638%
	6	I80.3 Phlebitis and thrombophlebitis of lower extremities, unspecified	3788	6.242%	1299	6.167%
	7	I80.8 Phlebitis and thrombophlebitis of other sites	2811	4.632%	350	1.662%
	8	I80.9 Phlebitis and thrombophlebitis of unspecified site	6977	11.497%	1505	7.145%
	9	I81 Portal vein thrombosis	389	0.641%	174	0.826%
	10	I82 Other venous embolism and thrombosis	8	0.013%	1	0.005%
	11	I82.0 Budd–Chiari syndrome	55	0.091%	12	0.057%
	12	I82.1 Thrombophlebitis migrans	11	0.018%	2	0.009%
	13	I82.2 Embolism and thrombosis of vena cava	63	0.104%	38	0.180%
	14	I82.3 Embolism and thrombosis of renal vein	84	0.138%	22	0.104%
	15	I82.8 Embolism and thrombosis of other specified veins	2867	4.725%	1538	7.302%
	16	I82.9 Embolism and thrombosis of unspecified vein	15,327	25.257%	6067	28.804%
	17	I63.6 Cerebral infarction due to cerebral venous thrombosis, nonpyogenic	66	0.109%	16	0.076%
	18	I67.6 Nonpyogenic thrombosis of intracranial venous system	50	0.082%	12	0.057%
	19	O22.2 Superficial thrombophlebitis in pregnancy	3	0.005%	-	0.000%
	20	O22.3 Deep phlebothrombosis in pregnancy	106	0.175%	2	0.009%
	21	O22.5 Cerebral venous thrombosis in pregnancy	3	0.005%	-	0.000%
	22	O87.1 Deep phlebothrombosis in the puerperium	126	0.208%	11	0.052%
	23	O87.3 Cerebral venous thrombosis in the peurperium	3	0.005%	2	0.009%
	24	G08 Intracranial and intraspinal phlebitis and thrombophlebitis	69	0.114%	20	0.095%
	25	G95 Other diseases of spinal cord	0	0.000%	0	0.000%
	26	K55.0 Acute vascular disorders of intestine	519	0.855%	223	1.059%
	27	K55.1 Chronic vascular disorders of intestine	178	0.293%	33	0.157%
		Total	48,412	79.779%	19,224	91.269%
Arterial TE	28	I21, I22, I24 Myocardial infarction and other acute ischemic heart disease	14	0.023%	2	0.009%
	29	I63 Cerebral infarction	54	0.089%	1	0.005%
	30	I74.0 Embolism and thrombosis of abdominal aorta	145	0.239%	44	0.209%
	31	I74.1 Embolism and thrombosis of other and unspecified parts of aorta	159	0.262%	51	0.242%
	32	I74.2 Embolism and thrombosis of arteries of upper extremities	163	0.269%	61	0.290%
	33	I74.3 Embolism and thrombosis of arteries of lower extremities	3	0.005%	-	0.000%
	34	I74.4 Embolism and thrombosis of arteries of extremities, unspecified	1614	2.660%	402	1.909%
	35	I74.5 Embolism and thrombosis of iliac artery	203	0.335%	74	0.351%
	36	I74.8 Embolism and thrombosis of other arteries	2757	4.543%	484	2.298%
	37	I74.9 Embolism and thrombosis of unspecified artery	6686	11.018%	478	2.269%
	38	N28.0 Ischemia and infarction of kidney	473	0.779%	242	1.149%
		Total	12,271	20.221%	1839	8.731%

Abbreviation: TE, thromboembolism.

## Data Availability

Data are available from the corresponding author upon reasonable request.

## Supplementary Materials

The following supporting information can be downloaded at: https://www.mdpi.com/article/10.3390/jcm11061477/s1, Table S1: Classification codes for type of each surgery; Table S2: Type of total claimed thromboembolism for each surgery; Table S3: Type of thromboembolism with anticoagulation treatment for each surgery; Table S4: Baseline characteristics of the whole cohort and those of each surgery group; Table S5: Multivariate analysis of variables associated with postoperative thromboembolism events.
