# Supplementary material for: Postoperative Thromboembolism According to the Type of Surgery: A Nationwide Study in the Republic of Korea
Source: J Clin Med. 2022 Mar 8;11(6):1477. doi: 10.3390/jcm11061477 (PMC8948856; doi:10.3390/jcm11061477)
Supplement: Supplementary file 1 [file jcm-11-01477-s001.zip › jcm-1606124-supplementary.pdf]

Supplementary Materials

Table S1. Classification codes for type of each surgery

No.	Surgery item	Classification code(s)
1	Cataract surgery	S5110, S5111, S5112, S5116, S5117 S5118, S5119
2	Tonsillectomy	Q2300, Q2310
3	Transluminal coronary angioplasty without insertion of stent	M6551, M6552, M6571, M6572
4	Transluminal coronary angioplasty with insertion of stent	M6561, M6562, M6563, M6564, *M6565, *M6566, *M6567
5	Coronary artery bypass graft	O1641, O1642, O1647, OA641, OA642 OA647, *O1640, *O1648, *O1649, *OA640, *OA648, *OA649
6	Cardiac catheterization	E7211, E7215, E7216, E7217, E7218 E7311, E7312, E7313, E7314, E7315 E7316, *E0720, *E0721,*E0722, *E0723, *E0724, *E0725, *E0726, *E0727, *E0728, *E0729
7	Operation for varicose vein	O0261, O0262, O0263, O0264, O0265, O0266, O0267, O2052
8	Appendectomy	Q2861, Q2862, Q2863
9	Operation for cardiac pacemaker	O0203, O0204, O0205, O0206, O0207, O0208, O0209, O0210, O2001, O2002, O2004, O2005
10	Cholecystectomy	Q7380, Q7410
11	Endoscopic or percutaneous biliary procedure	Q7761, Q7762, Q7763, Q7764, Q7765 Q7766, Q7767, Q7771, Q7772, Q7773 Q7774, Q7775, Q7776
12	Repair of inguinal hernia	Q7761, Q7762, Q7763, Q7764, Q7765 Q7766, Q7767, Q7771, Q7772, Q7773 Q7774, Q7775, Q7776
13	Transurethral prostatectomy	R3975, R3977
14	Open prostatectomy	R3950, R3960, RZ512
15	Hysterectomy	R4073, R4074, R4130, R4143, R4144, R4145, R4146, R4154, R4155, R4183, R4202, R4203, R4221, R4223, R4250, R4427, R4428, R4482
16	Cesarean section	R4514, R4516, R4517, R4518, R4519 R4520, R4507, R4508, R4509, R4510, R5001, R5002
17	Hip replacement	N0711, N1711, N0715, N1715, N2070, N2710, N3710, N4710
18	Total knee replacement	N2072, N2077
19	Partial excision of mammary gland	N7121, N7122, N7133, N7134, *N7136, *N7137
20	Total mastectomy	N7131, N7132, N7135, *N7130, *N7138, *N7139
21	Skull base surgery	S4801, S4802, S4803
22	Brain tumor surgery	S4633, S4634, S4635, S4636, S4637 S4743, *S4638, *S4639
23	Gastrectomy	Q0251, Q0252, Q0253, Q0254, Q0255 Q0256, Q0257, Q0258, Q2533, Q2534, Q2536, Q2537, Q2594 Q2598
24	Partial hepatectomy	Q7221, Q7222, Q7223, Q7224
25	Hepatic artery embolization	M6644
26	Thyroidectomy	P4551, P4552, P4553, P4554, P4558 P4561, P4565
27	Cardiac surgery (except coronary artery bypass graft)	O1671, O1672, O1680, O1701, O1702, O1703, O1704, O1705 O1710, O1711, O1721, O1723, O1740 O1750, O1760, O1770, O1781, O1782, O1783, O1791, O1792, O1793, O1794 O1795, O1796, O1797, O1798, O1800, O1810, O1821, O1822, O1823, O1824 O1825, O1826, O1840, O1841, O1850, O1861, O1873, O1874, O1875, O1878, O1879, O1960

28	Cleft lip and/or palate surgery	Q2161, Q2162, Q2191, Q2192, Q2193 Q2194
29	Operation for hemorrhoid	Q3012, Q3013, Q3014, Q3015, Q3016 Q3017
30	Endoscopic spine surgery	N1494
31	Spine surgery	N0303, N0444, N0445, N0446, N0447, N0451, N0452, N0453, N0466, N0468, N0469, N0471, N0472, N0473, N0474, N0480, N0500, N0630, N1491, N1492, N1493, N1495, N1496, N1497, N1498, N1499, N2461, N2462, N2463, N2464, N2465, N2466, N2467, N2468, N2469, N2470, N2471, N2472, N2491, N2492
32	Endoscopic sinus surgery	O0967, O0968, O1011, O1022, O1051 O1101, O1121, O1131, O1141, O1151 O1161, O1171, O1176, O1181
33	Sinus surgery	O0961, O0962, O0963, O0964, O0965 O0966, O1050, O1055, O1061, O1070 O1081, O1091, O1092, O1093, O1100 O1110, O1120, O1130, O1140, O1150 O1160, O1170, O1175, O1180

Note: The 2019 Statistical Information Report for Major Surgical Statistics provided by the National Statistical Office was used (http://kostat.go.kr/). The report consisted of data on 33 surgeries, including 16 surgeries of international interest and 17 surgeries reflecting domestic conditions. Among them, 26 surgeries that either required general anesthesia or were considered commonly performed procedures were selected for the present study. The areas painted in gray indicate the surgeries selected in this study.

Table S2. Type of total claimed thromboembolism for each surgery

	Type of thromboembolism*																																						
	1	2	3	4	5	6	7	8	9	10	11	12	13	14	15	16	17	18	19	20	21	22	23	24	25	26	27	28	29	30	31	32	33	34	35	36	37	38	Total
Total knee replacement	-	1	92	10	4,060	1,356	325	1,232	5	1	3	1	2	3	673	3,241	-	3	-	-	-	-	-	1	-	12	9	-	1	1	15	2	-	321	4	231	2,194	12	13,811
Hip replacement	-	-	24	11	1,635	428	100	474	15	-	2	1	3	4	458	1,249	1	2	-	-	-	-	-	3	-	18	3	-	2	10	2	4	-	101	7	109	293	16	4,975
Spine surgery	1	2	110	17	3,337	1,064	677	1,995	33	1	2	2	11	9	472	6,338	7	3	-	-	1	-	-	7	-	65	38	2	7	32	26	28	-	298	42	397	758	75	15,856
Coronary artery bypass graft	-	-	33	1	90	9	3	23	2	-	-	-	-	-	11	50	1	-	-	-	-	-	-	-	-	7	-	1	-	7	5	8	-	15	10	20	35	12	343
Partial hepatectomy	-	-	1	1	109	3	10	24	79	-	2	-	3	-	48	109	-	-	-	-	-	-	-	-	-	5	1	-	-	2	2	-	-	2	2	9	14	3	429
Cardiac surgery (except coronary artery bypass graft)	-	-	35	1	97	5	16	22	10	-	-	-	3	1	8	77	-	-	-	-	-	-	-	2	-	9	2	-	-	3	14	4	-	7	3	46	41	37	443
Gastrectomy	-	-	5	-	234	11	26	518	18	-	1	-	2	1	26	128	1	2	-	-	-	-	-	1	-	32	5	-	-	4	1	3	-	13	9	33	36	4	1,114
Hysterectomy	-	-	2	1	218	6	14	73	4	-	-	-	4	4	70	254	2	-	-	-	-	-	-	2	-	13	-	-	1	1	-	-	-	10	2	10	15	8	714
Endoscopic spine surgery	-	-	4	-	28	11	5	135	-	-	1	-	-	1	3	159	-	-	-	-	-	-	-	-	-	-	1	-	-	1	6	-	-	16	1	9	9	2	392
Brain tumor surgery	-	-	3	-	143	7	13	20	1	-	-	-	4	1	16	46	3	4	-	-	-	-	-	1	-	4	2	-	1	2	1	-	-	6	2	5	14	3	302
Open prostatectomy	-	-	4	-	20	-	3	13	1	-	-	-	-	-	6	11	-	-	-	-	-	-	-	-	-	-	1	-	-	-	-	-	-	2	1	7	11	1	81
Cholecystectomy	-	-	26	3	608	48	123	240	61	-	4	-	5	6	119	648	5	2	-	4	-	-	-	3	-	82	27	1	4	9	6	10	-	54	18	155	206	34	2,511
Transurethral prostatectomy	-	-	7	-	86	12	20	36	2	-	-	-	2	2	18	57	1	-	-	-	-	-	-	-	-	7	2	-	-	3	1	-	-	4	7	26	45	4	342
Skull base surgery	-	-	-	-	4	1	1	1	-	-	-	-	-	-	-	3	-	-	-	-	-	-	-	-	-	-	-	-	-	-	-	-	-	-	-	-	-	-	10
Cataract surgery	1	-	300	31	2,199	590	675	1,144	78	3	24	1	16	35	635	2,083	36	18	-	1	-	-	-	23	-	116	47	5	29	55	64	86	2	630	72	1,284	2,352	184	12,818
Total mastectomy	-	-	13	1	72	9	45	35	1	-	-	-	-	-	17	52	-	-	-	-	-	-	-	-	-	-	-	-	1	-	1	-	-	-	-	13	19	1	280
Repair of inguinal hernia	-	1	21	3	108	27	54	86	25	-	-	1	2	-	33	109	2	2	-	-	-	-	-	5	-	35	7	-	2	1	1	1	-	22	4	43	69	7	671
Partial excision of mammary gland	-	-	19	2	132	24	118	111	2	-	1	1	1	1	23	74	-	1	-	3	-	-	-	-	-	3	1	-	-	2	1	2	-	8	1	22	32	1	586
Appendectomy	-	1	30	2	159	27	145	153	18	-	3	1	1	4	59	144	2	-	-	8	-	4	-	2	-	69	12	1	2	3	3	3	-	21	2	38	89	18	1,024
Thyroidectomy	-	-	7	-	93	12	24	54	1	-	-	-	1	-	13	53	1	-	-	-	-	-	-	1	-	9	1	-	-	-	1	3	-	7	-	23	41	6	351
Sinus surgery	-	-	17	1	147	14	52	99	8	1	2	1	-	5	39	93	1	3	-	17	-	17	-	4	-	8	4	-	1	3	-	4	1	21	3	152	139	10	867
Endoscopic sinus surgery	-	-	24	1	88	22	67	113	7	-	3	1	-	3	22	63	-	3	-	1	-	-	-	12	-	8	5	1	-	-	2	2	-	17	-	36	71	6	578
Operation for hemorrhoid	-	-	90	1	165	77	215	181	9	1	4	1	2	4	72	198	1	3	-	4	-	4	-	2	-	13	9	3	3	4	7	3	-	35	5	78	184	23	1,401
Cesarean section	-	-	11	-	88	21	65	172	5	1	2	-	1	-	19	77	1	4	3	68	2	101	3	-	-	4	1	-	-	1	-	-	-	1	8	8	12	5	684
Cleft lip and/or palate surgery	-	-	-	-	-	-	-	-	-	-	-	-	-	-	-	-	-	-	-	-	-	-	-	-	-	-	-	-	-	-	-	-	-	-	-	-	2	-	2
Tonsillectomy	-	-	6	-	11	4	15	23	4	-	1	-	-	-	7	11	1	-	-	-	-	-	-	-	-	-	-	-	-	1	-	-	-	3	-	3	5	1	96
Total	2	5	884	87	13,931	3,788	2,811	6,977	389	8	55	11	63	84	2,867	15,327	66	50	3	106	3	126	3	69	-	519	178	14	54	145	159	163	3	1,614	203	2,757	6,686	473	60,681

Note: *Types of thromboembolism corresponding to the numbers are listed in Table 3.

Table S3. Type of thromboembolism with anticoagulation treatment for each surgery

	Type of thromboembolism*																																						
	**1**	**2**	**3**	**4**	**5**	**6**	**7**	**8**	**9**	**10**	**11**	**12**	**13**	**14**	**15**	**16**	**17**	**18**	**19**	**20**	**21**	**22**	**23**	**24**	**25**	**26**	**27**	**28**	**29**	**30**	**31**	**32**	**33**	**34**	**35**	**36**	**37**	**38**	**Total**
**Total knee replacement**	-	-	23	7	2,847	904	117	612	2	-	2	1	1	-	490	2,637	-	-	-	-	-	-	-	1	-	3	-	-	-	1	8	-	-	169	2	71	91	6	7,995
**Hip replacement**	-	-	9	9	1,178	165	51	297	11	-	1	1	3	1	345	855	-	1	-	-	-	-	-	2	-	6	1	-	-	6	1	2	-	67	5	53	43	11	3,124
**Spine surgery**	1	-	8	7	1,306	78	45	237	7	-	2	-	5	3	138	1,183	1	1	-	-	-	-	-	-	-	23	7	1	-	8	5	8	-	55	15	88	70	35	3,336
**Coronary artery bypass graft**	-	-	32	-	82	3	1	13	-	-	-	-	-	-	8	37	1	-	-	-	-	-	-	-	-	6	-	-	-	7	5	3	-	7	8	8	22	8	251
**Partial hepatectomy**	-	-	-	1	97	3	3	15	53	-	1	-	3	-	42	95	-	-	-	-	-	-	-	-	-	2	1	-	-	2	1	-	-	-	1	5	4	2	331
**Cardiac surgery (except coronary artery bypass graft)**	-	-	34	-	90	2	12	20	7	-	-	-	2	1	7	72	-	-	-	-	-	-	-	1	-	8	2	-	-	2	14	3	-	7	3	39	30	30	386
**Gastrectomy**	-	-	1	-	108	4	6	22	12	-	1	-	-	1	20	76	1	1	-	-	-	-	-	-	-	17	2	-	-	2	-	1	-	4	5	8	8	2	302
**Hysterectomy**	-	-	-	-	159	5	4	38	1	-	-	-	4	3	67	234	1	-	-	-	-	-	-	1	-	9	-	-	-	-	-	-	-	2	1	3	3	5	540
**Endoscopic spine surgery**	-	-	-	-	14	-	-	-	-	-	-	-	-	-	1	7	-	-	-	-	-	-	-	-	-	-	-	-	-	-	-	-	-	-	-	-	-	1	23
**Brain tumor surgery**	-	-	1	-	103	5	5	6	-	-	-	-	3	1	7	31	-	2	-	-	-	-	-	-	-	1	-	-	-	1	-	-	-	2	-	-	3	2	173
**Open prostatectomy**	-	-	2	-	14	-	-	2	-	-	-	-	-	-	2	2	-	-	-	-	-	-	-	-	-	-	-	-	-	-	-	-	-	-	1	-	-	-	23
**Cholecystectomy**	-	-	5	2	309	5	21	35	31	-	1	-	3	5	71	153	3	1	-	1	-	-	-	1	-	53	7	-	-	2	3	3	-	8	6	53	26	20	828
**Transurethral prostatectomy**	-	-	2	-	35	-	1	5	1	-	-	-	2	1	10	14	-	-	-	-	-	-	-	-	-	2	1	-	-	1	-	-	-	1	1	3	3	2	85
**Skull base surgery**	-	-	-	-	2	-	-	-	-	-	-	-	-	-	-	2	-	-	-	-	-	-	-	-	-	-	-	-	-	-	-	-	-	-	-	-	-	-	4
**Cataract surgery**	1	-	18	3	926	97	44	144	21	-	2	-	8	2	222	462	9	3	-	-	-	-	-	6	-	39	8	1	-	12	11	33	-	62	21	113	126	94	2,487
**Total mastectomy**	-	-	2	1	29	2	10	7	-	-	-	-	-	-	11	31	-	-	-	-	-	-	-	-	-	-	-	-	-	-	1	-	-	-	-	2	2	-	98
**Repair of inguinal hernia**	-	-	3	-	42	6	5	9	5	-	-	-	1	-	13	27	-	-	-	-	-	-	-	1	-	11	1	-	-	-	-	-	-	11	1	5	10	2	153
**Partial excision of mammary gland**	-	-	-	-	46	5	9	10	1	-	-	-	1	-	17	28	-	-	-	-	-	-	-	-	-	1	-	-	-	-	-	2	-	1	-	6	3	-	130
**Appendectomy**	-	-	4	-	96	4	8	11	11	-	1	-	-	2	23	40	-	-	-	-	-	-	-	1	-	29	3	-	-	-	1	1	-	2	1	9	7	9	263
**Thyroidectomy**	-	-	-	-	45	1	1	4	-	-	-	-	-	-	4	11	-	-	-	-	-	-	-	-	-	3	-	-	-	-	-	1	-	2	-	-	5	3	80
**Sinus surgery**	-	-	-	-	51	2	2	4	3	-	1	-	-	1	12	16	-	-	-	-	-	-	-	2	-	3	-	-	-	-	-	2	-	1	1	7	4	3	115
**Endoscopic sinus surgery**	-	-	1	1	34	1	1	1	3	-	-	-	-	-	8	11	-	1	-	-	-	-	-	4	-	-	-	-	-	-	-	1	-	1	-	4	7	3	82
**Operation for hemorrhoid**	-	-	2	-	63	7	4	8	3	-	-	-	1	1	13	30	-	-	-	-	-	-	-	-	-	6	-	-	1	-	1	1	-	-	2	5	7	3	158
**Cesarean section**	-	-	-	-	39	-	-	3	1	1	-	-	1	-	5	12	-	2	-	1	-	11	2	-	-	1	-	-	-	-	-	-	-	-	-	1	3	1	84
**Cleft lip and/or palate surgery**	-	-	-	-	-	-	-	-	-	-	-	-	-	-	-	-	-	-	-	-	-	-	-	-	-	-	-	-	-	-	-	-	-	-	-	-	1	-	1
**Tonsillectomy**	-	-	-	-	2	-	-	2	1	-	-	-	-	-	2	1	-	-	-	-	-	-	-	-	-	-	-	-	-	-	-	-	-	-	-	1	-	-	9
**Total**	2	-	147	5	1,373	125	84	203	49	1	4	-	12	6	330	669	9	6	-	1	-	11	2	14	-	93	12	1	1	12	14	41	-	80	26	153	175	118	3,660

Note: *Types of thromboembolism corresponding to the numbers are listed in Table 3.

Table S4. Baseline characteristics of the whole cohort and those of each surgery group

	Total patients, n	Age		Sex		History of cancer, n(%)	Atrial fibrillation or atrial flutter, n(%)	History of thromboembolism, n(%)	Antiplatelet or anticoagulant drug use between 2 weeks and 3 months before surgery, n(%)	Antiplatelet or anticoagulant drug use on the day of surgery, n(%)
		Mean	SD	Male, n(%)	Female, n(%)					
**Total knee replacement**	108,111	71.8	6.9	17,715(16.39)	90,396(83.61)	1,746(1.62)	3(0.003)	974(0.09)	21,152(19.57)	71,182(65.84)
**Hip replacement**	43,415	71.5	14.6	16,685(38.43)	26,730(61.57)	586(1.35)	1(0.002)	461(1.06)	8,684(20.00)	22,946(52.85)
**Spine surgery**	265,317	65.3	15.1	12,609(42.44)	52,708(57.56)	3,598(1.36)	9(0.003)	1,881(0.71)	42,685(16.09)	33,538(12.64)
**Coronary artery bypass graft**	6,591	64.9	11.6	5,057(76.73)	1,534(23.27)	121(1.84)	-	45(0.68)	4,914(74.56)	6,589(99.97)
**Partial hepatectomy**	9,322	61.8	11.6	6,308(67.67)	3,014(32.33)	1,101(11.81)	-	73(0.78)	1,469(15.76)	4,196(45.01)
**Cardiac surgery (except coronary artery bypass graft)**	10,296	41.6	29.7	5,315(51.62)	4,981(48.38)	99(0.96)	1(0.010)	87(0.84)	4,383(42.57)	10,030(97.42)
**Gastrectomy**	27,203	62.7	12.3	17,702(65.07)	9,501(34.93)	714(2.62)	1(0.004)	126(0.46)	3,614(13.29)	7,820(28.75)
**Hysterectomy**	20,831	54.5	11.8	1(0.00)	20,830(100)	579(2.78)	1(0.005)	80(0.38)	1,339(6.43)	4,338(20.82)
**Endoscopic spine surgery**	12,157	49.7	14.7	7,243(59.58)	4,914(40.42)	64(0.53)	-	30(0.25)	715(5.88)	167(1.37)
**Brain tumor surgery**	11,516	53.3	16.9	5,277(45.82)	6,239(54.18)	322(2.80)	-	42(0.36)	1,325(11.51)	5,008(43.49)
**Open prostatectomy**	3,387	69.6	6.3	3,387(100)	-	3,273(96.63)	-	25(0.74)	755(22.29)	1,753(51.76)
**Cholecystectomy**	129,081	55.7	15.6	63,202(48.96)	65,879(51.04)	3,964(3.07)	2(0.002)	617(0.48)	14,431(11.18)	12,744(9.87)
**Transurethral prostatectomy**	18,547	71.2	8.6	18,544(99.98)	3(0.02)	1,919(10.35)	-	119(0.64)	3,860(20.81)	952(5.13)
**Skull base surgery**	674	52.5	14.3	245(36.35)	429(63.65)	14(2.08)	-	2(0.30)	76(11.28)	262(38.87)
**Cataract surgery**	907,397	68.6	10.8	378,772(41.74)	528,625(58.26)	14,867(1.64)	24(0.003)	5,971(0.66)	160,729(17.71)	4,869(0.54)
**Total mastectomy**	22,091	45.4	16.4	7,628(34.53)	14,463(65.47)	453(2.05)	-	41(0.19)	1,070(4.84)	2,431(11.00)
**Repair of inguinal hernia**	56,698	49.6	27.6	49,873(87.96)	6,825(12.04)	2,118(3.74)	-	241(0.43)	6,197(10.93)	653(1.15)

**Partial excision of mammary gland**	63,033	48.3	12.8	345(0.55)	62,688(99.45)	1,804(2.86)	-	119(0.19)	2,294(3.64)	1,488(2.36)
**Appendectomy**	137,344	39	19.9	73,537(53.54)	63,807(46.46)	942(0.69)	-	233(0.17)	5,200(3.79)	1,527(1.11)
**Thyroidectomy**	51,506	48.9	13.3	13,006(25.25)	38,500(74.75)	35,361(68.65)	1(0.002)	97(0.19)	2,442(4.74)	1,609(3.12)
**Sinus surgery**	158,677	41.2	17.8	72,132(45.46)	86,545(54.54)	1,214(0.77)	1(0.001)	357(0.22)	6,205(3.91)	536(0.34)
**Endoscopic sinus surgery**	109,454	40.7	17.4	74,926(68.45)	34,528(31.55)	773(0.71)	-	158(0.14)	4,585(4.19)	1,049(0.96)
**Operation for hemorrhoid**	303,456	45.6	15.0	162,403(53.52)	141,053(46.48)	2,299(0.76)	4(0.001)	476(0.16)	10,970(3.62)	297(0.10)
**Cesarean section**	255,600	33.3	4.4	-	255,600(100)	1,389(0.54)	-	143(0.06)	1521(0.60)	593(0.23)
**Cleft lip and/or palate surgery**	1,282	5.71	11.9	682(53.20)	600(46.80)	4(0.31)	-	1(0.08)	10(0.78)	6(0.47)
**Tonsillectomy**	66,307	20.1	15.2	37,875(57.12)	28,432(42.88)	165(0.25)	-	21(0.03)	369(0.56)	162(0.24)
**Total**	2,799,293	55	20	1,150,469 (41.10)	1,648,824 (58.90)	79,489 (2.84)	48 (0.002)	12,420 (0.44)	310,994 (11.11)	196,745 (7.03)

Abbreviations: SD, standard deviation

Table S5. Multivariate analysis of variables associated with postoperative thromboembolism events

Variables	Total claimed thromboembolism, n(%)		Thromboembolism with anticoagulant treatment, n(%)	
	OR (95% CI)	p value	OR (95% CI)	p value
Age	1.014 (1.013, 1.015)	<0.001	1.016 (1.015, 1.018)	<0.001
Female vs. male	0.907 (0.887, 0.926)	<0.001	0.907 (0.875, 0.941)	<0.001
History of cancer vs. no	1.186 (1.109, 1.267)	<0.001	1.303 (1.177, 1.444)	<0.001
Atrial fibrillation or atrial flutter vs. no	2.146 (0.506, 9.103)	0.300	3.698 (0.462, 29.635)	0.218
History of thromboembolism vs. no	>999 (<0.001, >999)	1.000	49.578 (46.445, 52.923)	<0.001
Antiplatelet or anticoagulant drug use between 2 weeks and 3 months before surgery vs. no use	0.883 (0.86, 0.906)	<0.001	0.752 (0.723, 0.781)	<0.001
Antiplatelet or anticoagulant drug use on the day of surgery vs. no use	9.851 (9.569, 10.14)	<0.001	82.653 (78.771, 86.726)	<0.001
Prophylactic anticoagulant treatment vs. no use	0.052 (0.05, 0.054)	<0.001	0.030 (0.028, 0.031)	<0.001
Type of surgery vs. reference category				
Total knee replacement	9.641 (8.412, 11.051)	<0.001	5.516 (4.356, 6.984)	<0.001
Hip replacement	7.990 (6.958, 9.175)	<0.001	6.018 (4.745, 7.631)	<0.001
Spine surgery	4.887 (4.271, 5.592)	<0.001	2.052 (1.621, 2.597)	<0.001
Coronary artery bypass graft	7.695 (6.406, 9.242)	<0.001	6.901 (5.242, 9.086)	<0.001
Partial hepatectomy	3.876 (3.261, 4.607)	<0.001	5.871 (4.498, 7.662)	<0.001
Cardiac surgery (except coronary artery bypass graft)	7.932 (6.648, 9.463)	<0.001	9.334 (7.16, 12.167)	<0.001
Gastrectomy	3.807 (3.282, 4.415)	<0.001	2.111 (1.621, 2.748)	<0.001
Hysterectomy	3.793 (3.24, 4.44)	<0.001	9.437 (7.309, 12.184)	<0.001
Endoscopic spine surgery	3.669 (3.094, 4.351)	<0.001	0.87 (0.537, 1.41)	0.5726
Brain tumor surgery	3.17 (2.639, 3.807)	<0.001	4.634 (3.485, 6.161)	<0.001
Open prostatectomy	1.604 (1.184, 2.172)	0.002	0.935 (0.565, 1.549)	0.795

Cholecystectomy	1.533 (1.331, 1.766)	<0.001	1.49 (1.167, 1.901)	0.001
Skull base surgery	1.754 (0.86, 3.576)	0.122	1.869 (0.595, 5.867)	0.284
Cataract surgery	0.742 (0.648, 0.849)	<0.001	1.098 (0.867, 1.39)	0.438
Total mastectomy	1.456 (1.208, 1.754)	<0.001	2.058 (1.503, 2.818)	<0.001
Repair of inguinal hernia	0.873 (0.741, 1.029)	0.105	1.339 (1.007, 1.78)	0.044
Partial excision of mammary gland	0.992 (0.842, 1.168)	0.922	1.229 (0.913, 1.655)	0.174
Appendectomy	0.816 (0.7, 0.95)	0.009	1.323 (1.014, 1.726)	0.039
Thyroidectomy	0.561 (0.465, 0.677)	<0.001	0.659 (0.472, 0.921)	0.0147
Sinus surgery	0.446 (0.38, 0.524)	<0.001	0.418 (0.308, 0.568)	<0.001
Endoscopic sinus surgery	0.523 (0.443, 0.617)	<0.001	0.484 (0.351, 0.669)	<0.001
Operation for hemorrhoid	0.403 (0.347, 0.468)	<0.001	0.387 (0.292, 0.513)	<0.001
Cesarean section	0.356 (0.303, 0.419)	<0.001	0.358 (0.259, 0.494)	<0.001
Cleft lip and/or palate surgery	0.177 (0.025, 1.264)	0.0843	0.983 (0.133, 7.264)	0.9863
Tonsillectomy	0.21 (0.161, 0.274)	<0.001	0.161 (0.08, 0.323)	<0.001

Abbreviations: OR, odds ratio; CI, confidence interval
